# Zika virus NS2A inhibits interferon signaling by degradation of STAT1 and STAT2

**DOI:** 10.1080/21505594.2021.1935613

**Published:** 2021-08-02

**Authors:** Elisa Fanunza, Fabrizio Carletti, Marina Quartu, Nicole Grandi, Laura Ermellino, Jessica Milia, Angela Corona, Maria Rosaria Capobianchi, Giuseppe Ippolito, Enzo Tramontano

**Affiliations:** a Department of Life and Environmental Sciences, University of Cagliari, Monserrato, Italy; b Laboratory of Virology, National Institute for Infectious Diseases, L.Spallanzani͟ IRCCS, Rome, Italy; c Department of Biomedical Sciences, University of Cagliari, Monserrato, Italy

**Keywords:** Zika virus, Ns2a, interferon, Ifn signaling, Ifn evasion, stat1, stat2, degradation, phosphorylation

## Abstract

The Interferon (IFN) response is crucial to restrain pathogenic infections. Investigations into flavivirus-host interactions reported that the high virulence is linked to innate immune evasion. Zika Virus (ZIKV) has developed diversified strategies to evade the innate immune system. We report that the viral protein NS2A counteracts the IFN response by strongly suppressing the IFN signaling. NS2A targets transcription factors STAT1 and STAT2, to impede their nuclear localization, thereby suppressing the transcription of ISRE promoter and IFN-stimulated genes. We found that NS2A promotes degradation of STAT1 and STAT2. Treatment of NS2A transfected cells with MG132 restores the levels of both transcription factors, suggesting the involvement of the proteasome system. Given the impact that the IFN antagonism has on flavivirus virulence, the knowledge gained by characterizing the mechanism through which ZIKV evades the IFN response paves the ground for new strategies to attenuate the pathogenesis and to develop countermeasures against effective pharmacological targets.

## Introduction

Zika virus (ZIKV) is an arthropod-borne virus that belongs to the family of *Flaviviridae*, firstly isolated in 1947 in Uganda [1,2]. ZIKV has been described as causing sporadic human infections [3], until 2015 when it was declared a global public health concern, due to its association with microcephaly cases and other severe neurologic disorders [4,5].

Like the other flaviviruses, ZIKV has a positive-sense, single-stranded RNA genome (ssRNA+) of approximately 10.7 kb in length, which is in complex with multiple copies of the capsid protein. The genome encodes a single polyprotein that is processed by viral and cellular proteases into three structural proteins, the capsid (C), membrane (prM) and envelope (E) proteins that mediate virus attachment, entry and encapsidation, and seven non-structural (NS) proteins (NS1, NS2A, NS2B, NS3, NS4A, NS4B and NS5), which function in viral replication and polyprotein processing [6].

The type I Interferon (IFN-α/-β) signaling pathway is a potent cellular antiviral response that occurs early during a viral infection [7,8]. After IFN-α/-β binding to the heterodimeric IFN-α receptor (IFNAR), the tyrosine kinases JAK1 and TYK2 promote the phosphorylation of STAT1 and STAT2, respectively [9,10]. In the canonical IFN I response, phosphorylated STAT1 (P-STAT1) forms a homodimer or a hetero-trimeric complex with phosphorylated STAT2 (P-STAT2) and the IFN-regulatory factor 9 (IRF9), named IFN-stimulated gene factor 3 (ISGF3). Entering into the nucleus, the complex recognizes the promoter of the IFN Stimulated Genes (ISGs), termed IFN-stimulated response elements (ISRE), leading to the activation of the cellular antiviral state [11,12]. Besides the canonical pathway, a number of non-canonical interferon pathways and regulators have also been discovered [13]. The cascade activated in response to type II IFN differs in some respects from type I IFN pathway. The dimerization of the IFN-γ receptor leads to the recruitment of JAK1 and JAK2 that subsequently phosphorylate STAT1 at tyrosine in position 701, P-STAT1 dimerizes forming a STAT1–STAT1 homodimer, that translocates into the nucleus and binds to the gamma-activation sequence (GAS), stimulating transcription of ISGs [7].

The IFN system plays a critical but controversial role in orchestrating protection against ZIKV. To limit ZIKV infection, placental macrophages and primary human trophoblasts produce type I and III IFNs [14–19]. Moreover, the infection of human fibroblasts results in the increased expression levels of components of the IFN production cascade, such as RIG-I, MDA-5, TLR3, IRF7, as well as ISGs, such as ISG15, OAS2 and MX1. The expression of members of the IFITM family and viperin has been shown to inhibit ZIKV replication. Furthermore, ZIKV infection has been demonstrated to be restricted in mice by type I IFNs. Especially, viral replication and pathogenicity are accentuated in mice lacking IFNAR, STAT2, MAVS or IRF transcription factors [20].

On the contrary, in human monocyte-derived dendritic cells and skin fibroblasts, the treatment with type I IFN after or in concomitance with ZIKV infection partially reduces the viral replication, suggesting viral counteraction effects of type I IFNs [21]. This impaired ability of type I IFN to restrict infection has been demonstrated to be dependent on the viral antagonism of IFN-mediated phosphorylation of STAT1 and STAT2 [21]. Several recent studies have reported the ability of ZIKV to suppress the IFN and ISGs expression in ZIKV-infected cells *in vitro* leading to the significant reduction in type I and type III IFN signaling [21–25]. A recent study reported the ability of the ZIKV protease complex, NS2B3, to suppress the IFN signaling pathway. NS2B3 has been shown to interact with JAK1 promoting its degradation, resulting in the inhibition of STAT1 phosphorylation and in the reduced expression of ISGs such as ISG15, IFIT1, IFIT2 and viperin in NS2B3 overexpression cells upon stimulation with IFNβ [24]. The JAK/STAT cascade is targeted also by ZIKV NS5, which has been demonstrated to bind and degrade STAT2 [22,26].

NS2A is a small protein (20 kDa), associated with the endoplasmic reticulum, implicated in the modulation of the host IFN response in different flaviviruses. NS2A is responsible for the suppression of IFN-β transcription, being an important determinant of virulence in Kunjiin Virus (KUNV) [27,28]. In Japanese Encephalitis Virus (JEV), it acts blocking the cellular protein PKR [29]. ZIKV NS2A is also known to block the IFN production [22,23,30]. However, its effects on IFN signaling have not been yet investigated. This study aims to determine whether ZIKV NS2A also exerts effective inhibitory function on the IFN signaling system. Our findings demonstrate that ZIKV NS2A inhibits both the type I and type II IFN downstream effects by blocking JAK-STAT cascade through the degradation of cellular STAT1 and STAT2. Our study provides evidence of a new role for NS2A in antagonizing innate host antiviral defense to support successful ZIKV infections.

## Materials and methods

### Cells and Reagents

HEK293T (ATCC) or Vero (ATCC) cells were grown in Dulbecco’s modified Eagle’s medium (Gibco) supplemented with 10% fetal bovine serum (Gibco) and 1% penicillin/streptomycin (Sigma). Cells were grown at 37°C in a humidified 5% CO_2_ atmosphere. Plasmid pISRE-luc was a kind gift of Prof Ian Goodfellow (University of Cambridge, UK). Plasmid for FLAG-VP24 was kindly given by Marco Sgarbanti (Istituto Superiore di Sanità, Italy). pRL-TK was purchased from Promega. T-Pro P-Fect Transfection Reagent was from T-Pro Biotechnology. Human Recombinant IFN-α was purchased from Thermo Fisher Scientific. Mouse monoclonal anti-FLAG M2 was obtained from Sigma. Rabbit antibodies against P-STAT1, P-STAT2, P-JAK1, P-TYK2, STAT2, GAPDH and the anti-rabbit HRP-linked IgG were purchased from Cell Signaling. Rabbit anti-STAT1, anti-mouse HRP-linked IgG, goat anti-mouse IgG Alexa Fluor 488, goat anti-rabbit IgG Alexa Fluor 594 and Pierce ECL Western Blotting Substrate were from Thermo Fischer Scientific.

### Virus stock preparation

For virus stock preparation, Vero E6 cells were infected with ZIKV 2016/INMI1 (INMI1) isolate (GenBank Accession number KU991811) obtained from a traveler returning from Brazil in January 2016. Cell lysates were clarified, aliquoted, and stored at −80°C until use. Virus titration was performed on Vero E6 cell line by limiting dilution assay; the titer was calculated using the method of Reed and Muench and expressed as tissue culture infectious-dose TCID_50_/mL. Virus stock titers were 10^6.12^ TCID_50_/mL.

### Construction of mammalian expression plasmids

Each ZIKV NS gene was amplified from the strain Brazil/ 2016/INMI1 (GenBank: KU991811). The coding sequences with a C-terminal Flag tag were subcloned into the pcDNA3.1 (+) using NheI and EcoRI as restriction enzymes. Our library included the non-structural proteins: NS1, NS2A and the fusion proteins NS2B/3 and NS3/4A. The primer pairs used for subcloning are listed in Table 1.

**Table 1.** Primers for subcloning

Primer name^a^	Sequence (5ʹ to 3ʹ)
NS1_F NS1_R NS2/B3_F NS2/B3_R NS3/4A_F NS3/4A_R NS2A_1-226__F NS2A_1-226__R NS2A_12-226__F NS2A_12-226__R NS2A_51-226__F NS2A_51-226__R NS2A_1-50__F NS2A_1-50__R NS2A_51-100__F NS2A_51-100__R NS2A_101-150__F NS2A_101-150__R NS2A_151-200__F NS2A_151-200__R NS2A_201-226__F NS2A_201-226__R	GTGGGGTGTTCGGTGGACTTC AAGGGAGAAGTGATCCATGTG CCTAGCGAAGTACTCACAGCT AGCCGCTCCTCTTTTCCCAGC ACCACAGATGGAGTGTACAGA TCTTTGCTTTTCTGGCTCAGG GGATCAACTGATCACATGGAT CCGCTTCCCACTCCTTGTGAG GGAGTGCTTGTGATTCTGCTC CCGCTTCCCACTCCTTGTGAG ATGAGTGACCTGGCTAAGCTT CCGCTTCCCACTCCTTGTGAG GGATCAACTGATCACATGGAT TGAAAATCCTCCCAGGATCAT ATGAGTGACCTGGCTAAGCTT TGTCCAATTAGCTCTGAAGAT CCCCGTGAAAGCATGCTGCTG GATGTTGTCAGTGCGTGGAAC ACCTTGGCAATCCTGGCTGCT GGCCATGACAAATGGTAAGTT CTGGGACTAACCGCTGTGAGG CCGCTTCCCACTCCTTGTGAG

^a^F, forward; R, reverse.

## Luciferase reporter Gene Assays

HEK293T or Vero cells (1.5 × 10^4^ cells/well) were seeded in 96-well plates and transfected after 24 h. pIFNβ-luc, pISRE-luc, or pGAS-luc were used to quantify IFNβ production, IFN-I and IFN-II signaling, respectively [31–33]. Reporter vectors were cotransfected with pRL-TK and empty vector or ZIKV NS proteins expression plasmids and were incubated with T-Pro P-Fect Transfection Reagent (RT) in reduced serum medium Optimem (Gibco) for 20 min at room temperature. Transfection complexes were then gently added into individual wells. After 24 h, cells were stimulated with viral Influenza Virus RNA for 24 h, IFN-α for 8 h or IFN-γ for 24 h to test IFNβ production, IFN-I and IFN-II signaling, respectively. Then, cells were lysed with harvesting buffer (50 mM Na-MES [pH 7.8], 50 mM Tris-HCl [pH 7.8], 1 mM dithiothreitol, and 0.2% Triton X-100). To lysates, luciferase assay buffer (125 mM Na-MES [pH 7.8], 125 mM Tris-HCl [pH 7.8], 25 mM magnesium acetate, and 2.5 mg/mL ATP) and 1 mM D-luciferin were added. Firefly luminescence was measured in Victor3 luminometer (Perkin Elmer). Then, coelenterazine assay buffer (125 mM Na-MES [pH 7.8], 125 mM Tris-HCl [pH 7.8], 25 mM magnesium acetate, 5 mM KH_2_PO_4_, 10 μM coelenterazine) was added and the luminescence was read. Relative light units (RLU) were normalized as the fold induction over unstimulated controls. Luciferase activity was expressed as the percentage of promoter expression in viral proteins transfected cells normalized to empty vector cells (100% of activation). Each assay was performed in triplicate.

### IC_50_ calculations

To determine the concentration of ZIKV NS2A plasmid required to inhibit 50% of promoter expression (IC_50_), we used the log agonist concentration versus response, variable slope algorithm in GraphPad Prism software where:

(1) $$Y=Bottom+\left(Top-Bottom\right)/\left(1+10\left(\left(LogIC50\right)\right)\right)$$

IC_50_ values were calculated based on three independent experiments performed in triplicate.

### Co-immunoprecipitation experiment

HEK293T cells were transfected with empty vector or expression plasmid for FLAG-NS2A and lysed in PBS containing 1.5% Triton X-100, 1 mM Na_3_VO_4_, 1 mM DTT and 1x cOmplete protease inhibitor cocktail (Roche). Lysates were incubated with anti-FLAG M2 magnetic beads overnight at 4°C. Precipitated proteins were eluted by boiling with SDS sample loading buffer. Whole-cell lysates and immunoprecipitated samples were analyzed by western blot as previously described [34]. Membranes were probed with anti-FLAG M2 (1 μg/mL), anti-STAT1-tot (1:1000) and anti-GAPDH (1:1000). Secondary antibodies anti-mouse (1:10,000) and anti-rabbit (1:2000) HRP-linked IgG were used. Images were captured with Chemidoc MP Imaging System (Bio-Rad).

### Immunoblot analysis

To detect protein expression levels, HEK293T cells were seeded in 6-well plates (4 x 10^5^ cell/mL) and transfected with empty vector or ZIKV FLAG-NS2A plasmid. 36 h post-transfection, the medium was replaced with IFN-α. Thirty minutes post-IFN addition, cells were harvested in cold PBS containing 1.5% Triton X-100, 1 mM Na_3_VO_4_, 1 mM DTT and 1x cOmplete protease inhibitor cocktail (Roche). Total cell extracts were analyzed by sodium dodecyl sulfate-polyacrylamide gel electrophoresis (SDS-PAGE) and then transferred to a polyvinylidene difluoride (PVDF) membrane. Membranes were blocked with 3% nonfat dry milk in TBS (50 mM Tris-HCl, 0.138 M NaCl, 2.7 mM KCl, pH 8.0) and probed with rabbit primary antibodies, P-JAK1 (1:1000), P-TYK2 (1:1000), P-STAT1 (1:1000), STAT1-tot (1:1000), P-STAT2 (1:1000), STAT2-tot (1:1000) and GAPDH (1:1000) for normalization. Secondary antibodies were HRP-linked anti-rabbit IgG (1:2000). Detection was performed using Pierce ECL Western Blotting Substrate and Chemidoc MP Imaging System (Biorad). Bands quantification was performed using Image Lab software calculating the average between three independent experiments.

### Immunofluorescence

For immunostaining, HEK293T cells were seeded in 6-well plates (3 x 10^5^ cell/well) and cotransfected with Lipofectamine 3000 transfection reagent (Invitrogen) with 2.5 μg/well of pcDNA3.1 or plasmids for ZIKV proteins. After 36 h of transfection, cells were treated with IFN-α for 30 min and then fixed with 4% paraformaldehyde in PBS for 15 min at RT. Permeabilization was performed with ice-cold 100% methanol for 10 min, membranes were washed three times for 5 minutes with PBS, and incubated in blocking buffer (PBS containing 0.2% Triton X-100, 10% normal goat serum and 3% bovine serum albumin) for 1 h at RT. The cells were then stained for primary antibodies: anti-Flag (1:500), anti-P-STAT1 (1:400), anti-P-STAT2 (1:200), anti-STAT1 (1:100) for 1 h. After washes with PBS, cells were incubated with secondary antibodies conjugated to Alexa Fluor 488 and Alexa Fluor 594 (1:500) for 1 h and then 1 min with Hoechst (Thermo Fischer Scientific). Stained cells were mounted on glass slides with Glycerol Mounting Medium with DABCO as anti-fade reagent and observed with an Olympus BX61 microscope, equipped with epifluorescence illumination, and digital images were captured with a Leica DF 450 C camera. The cultures were examined at 40x magnification. Cells count was performed using the Cell Counter plugin image analysis program ImageJ.

### RNA extraction and quantitative real-time PCR

To determine the effect of NS2A on the expression of ISGs, ISG15 and OAS1, HEK293T cells in 6-well plates (3 x 10^5^ cell/well) were transfected with 2.5 μg/well of empty vector or expression plasmids for ZIKV proteins using Lipofectamine 3000 transfection reagent (Invitrogen). After 36 h, the cells were treated with IFN-α (10 ng/mL) for 24 h. Total RNA was extracted from transfected cells with TRIzol reagent (Invitrogen) as previously described [35]. Luna Universal One-Step RT-qPCR kit (New England Biolabs) was used to reverse transcribed and amplified mRNA. Quantitative real-time PCR (RT-qPCR) experiments were performed in triplicate. mRNA expression levels were normalized to the level of GAPDH. Results are shown as folds induction of stimulated over not stimulated samples. All qPCR primers used in this study are listed in Table 2.

**Table 2.** Primers used for RT-qPCR

Primer name^a^	Sequence (5ʹ to 3ʹ)
GAPDH_F GAPDH_R ISG15_F ISG15_R OAS1_F OAS1_R	GAGTCAACGGATTTTGGTCGT TTGATTTTGGAGGGATCTCG TCCTGGTGAGGAATAACAAGGG CTCAGCCAGAACAGGTCGTC AGCTTCATGGAGAGGGGCA AGGCCTGGCTGAATTACCCAT

^a^F, forward; R, reverse.

### Multiple alignments

To design primers of ZIKV NS proteins, pairwise and multiple alignments of amino acids sequences of ZIKV isolate Brazil/2016/INMI1 (GenBank: KU991811.1), ZIKV MR766 (GenBank: LC002520.1), ZIKV Ganxian (GenBank: KU744693.1), DENV-1 (NCBI: NC_001477.1), YFV (NCBI: NC_002031.1) and WNV (NCBI: NC_009942.1) were generated with Geneious bioinformatics software platform, version 8.1.4 [36] using MAFFT algorithm FFT-NS-i x1000 [37] with default parameters.

Using Clustal Omega [38], ZIKV isolate Brazil/2016/INMI1 NS2A fragments were aligned with NS2As of representative Flaviviridae: DENV (sp|P14340|1128-1345), WNV (sp|P06935| 1140–1370), TBEV (sp|P14336|1129-1358), YFV (sp|P03314|1131-1354) and JEV (sp|P32886|1147-1373). NS2A KUNV were form KUNV strain MRM61C.

## Phylogenetic analysis

The phylogenetic tree was built from manually optimized multiple alignments using Mega Software, version 6 [39] and neighbor joining statistical method. Amino acid sequences NJ trees were built using the Poisson model and applying pairwise deletion option. Phylogenies were tested by the bootstrap method with 1000 replicates. All amino acid sequences were retrieved from Uniprot database.

### Graphics

Graphics of experiments were performed using GraphPad Prism software 6.01 (GraphPad software, Inc, 2012).

## Results

### Effect of ZIKV proteins on the IFN-α-induced ISRE expression

**Figure 1.**
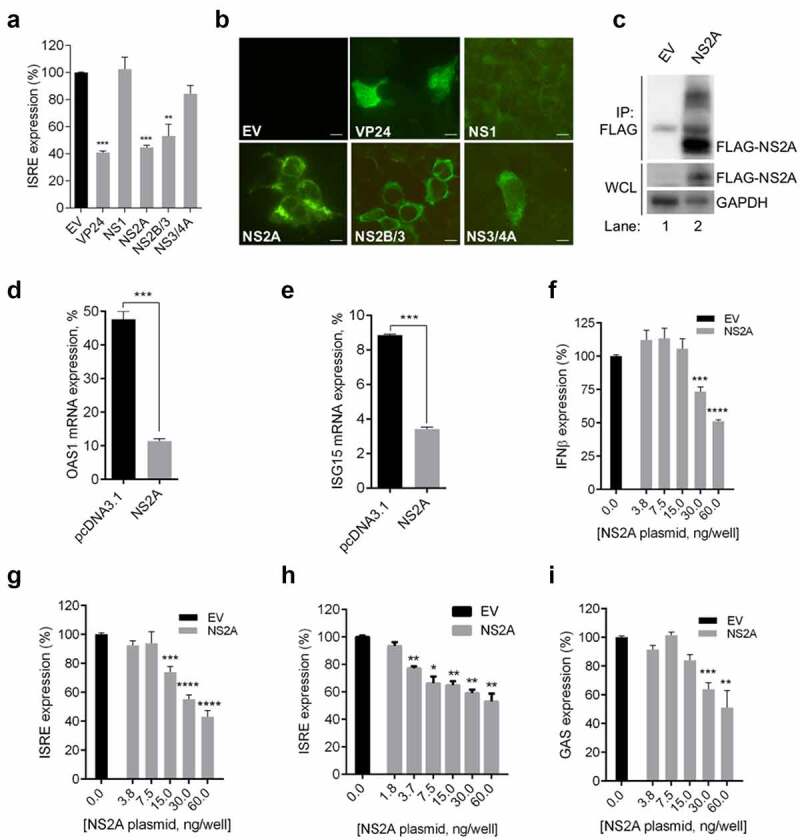
**ZIKV NS2A inhibits IFN production and IFN signaling responses**. (a) Percentage of ISRE expression in HEK293T cells transfected with empty vector (EV) or expression plasmids for EBOV VP24/ZIKV proteins and stimulated with IFN-α. (b) Immunofluorescence of HEK293T cotransfected with empty vector (EV) or FLAG-tagged expression plasmids for EBOV VP24/ZIKV proteins. Cells were stained for antibody to FLAG. Scale bar, 10 μm. (c) Immunoprecipitation of FLAG-tagged NS2A using anti-FLAG magnetic beads (IP:FLAG). Immunoprecipitated proteins were detected by Western blotting with antibodies to FLAG. The presence of transfected protein in whole cell extracts (WCE) was verified by detection with the corresponding antibody. (d) Evaluation of OAS1 transcript levels in cells transfected with empty vector (EV) or ZIKV NS2A in presence of IFN-α. (e) Evaluation of ISG15 transcript levels in HEK293T cells transfected with empty vector (EV) or ZIKV NS2A in presence of IFN-α. (f) Percentage of pIFNβ-luc expression in HEK293T cells transfected with increasing concentrations of ZIKV NS2A after stimulation with IAV vRNA. (g) Percentage of pISRE-luc expression in HEK293T cells transfected with increasing concentrations of ZIKV NS2A after stimulation with IFN-α. (h) Percentage of pISRE-luc expression in Vero cells transfected with increasing concentrations of ZIKV NS2A after stimulation with IFN-α. (i) Percentage of pGAS-luc expression in HEK293T cells transfected with increasing concentrations of ZIKV NS2A after stimulation with IFN-γ

With the aim of identifying ZIKV proteins able to inhibit the IFN-signaling pathway, we prepared a library of expression plasmids for the ZIKV individual proteins (NS1, NS2A, NS2B/3 and NS3/4A) and tested them for their ability to suppress the IFN-α-induced ISRE-driven transcription in a dual-luciferase reporter assay [33]. Briefly, HEK293T were cotransfected with pISRE-luc, RL-TK and each plasmid for the ZIKV proteins, and subsequently stimulated with IFN-α. As positive control of inhibition, we used EBOV VP24 and ZIKV NS2B/3, known inhibitors of the JAK/STAT cascade [24,40]. Results showed that, as expected, transfection with empty vector had no effect on IFN-α-induced ISRE-driven transcription; by contrast, transfection with the positive controls VP24 and NS2B/3 led to a high suppression of the luciferase reporter activity. Notably, substantial suppression of IFN-α-induced ISRE expression was also observed in cells expressing NS2A protein, while it was not observed in cells expressing the NS1 or NS3/4A proteins (Figure 1(a)). The expression of each viral protein was confirmed by immunofluorescence (Figure 1(b)).

(A, D-I) Significance calculated using atwo-tailed unpaired Student’s t-test, *P < 0.05, **P< 0.01, ***P< 0.001, ****P< 0.0001. Error bars indicate the mean ± SD. Data from at least 3 independent experiments.

In order to further validate the expression of NS2A, we performed immunoprecipitation analysis of HEK293T cell lysates transfected with ZIKV FLAG-tagged NS2A. We observed the expression of an immunoreactive protein with the same molecular weight of NS2A, 22 KDa, hence confirming that over-expression of NS2A was induced in transfected cells. As expected, NS2A was not detectable in empty vector transfected cells (Figure 1(c)).

To further confirm the inhibitory effect of NS2A on ISRE transcription using a different end-point, mRNA expression levels of two common ISGs, ISG15 and OAS1, were detected by qRT-PCR in HEK293T cells cotransfected with NS2A. Results showed that the mRNA levels of ISG15 and OAS1 induced by IFN-α were significantly reduced in cells cotransfected with NS2A, as compared to empty vector cotransfected cells (Figure 1(d,e)).

The antagonistic effect of ZIKV NS2A on IFN-β production has been already reported [22,23,30]. To investigate whether NS2A was more effective inhibiting the IFN production or induction cascade, we evaluated the protein effect on viral RNA-induced IFN-β promoter transcription and the IFN-α-induced ISRE transcription by luciferase assays [31,33]. Thus, by means of a dose–response curve obtained by cotransfecting cells with increasing concentration of NS2A (3.8–60 ng/well) and calculated the EC_50_ values; we found that NS2A was able to dose-dependently suppress both cascades, showing EC_50_ values of 60 ng/ well (Figure 1(f)) and 30 ng/well (Figure 1(g)) for IFN-β and ISRE inhibition, respectively, suggesting a higher potency in inhibiting the IFN signaling system.

Moreover, to confirm the specific NS2A inhibition of exogenous IFN-α-induced ISRE transcription, we performed a dose–response curve in Vero cells, which can respond to IFN but do not produce it [41]. In these cells, luciferase activity is the specific result of exogenously added IFN. In this system, we observed that NS2A was still able to exert its inhibitory effect on ISRE expression, confirming a not confined cell-type effect (Figure 1(h)).

Finally, we asked whether NS2A is also able to inhibit the IFN-γ-mediated gene expression. With this aim, IFN-γ-induced GAS promoter activation status in NS2A-contrasfected cells was evaluated. HEK293T cells were transfected with pGAS-luc plasmid and increasing concentration of NS2A and then stimulated with IFN-γ. The dose-dependent assay demonstrated that NS2A proteins inhibited also the GAS promoter activation in a dose-dependent manner. Overall, these results showed that transfection with NS2A resulted in the inhibition of the IFN-α stimulation of ISRE promoter more potent than the inhibition of the IFN-γ stimulation of the GAS promoter (EC_50_ value: 60 ng/well) (Figure 1(i)).

### NS2A Inhibits STAT1 Phosphorylation and Mediates its Degradation

**Figure 2.**
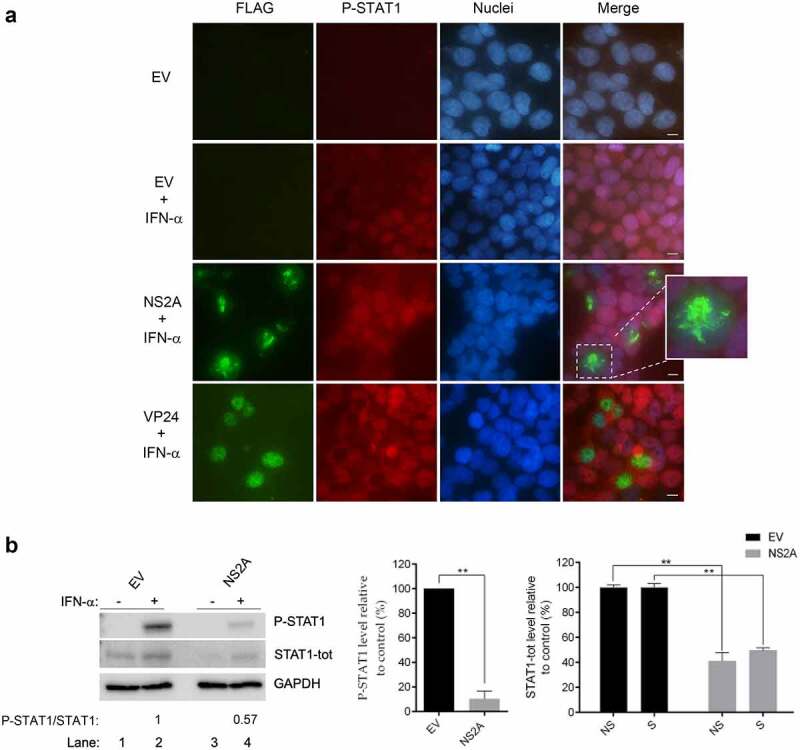
**Inhibition of STAT1 phosphorylation mediated by ZIKV NS2A**. (a) Immunofluorescence of HEK293T transfected with empty vector (EV) and stimulated (row 2) or not (row 1) with IFN-α, transfected with ZIKV NS2A (row 3) or EBOV VP24 (row 4) and stimulated with IFN-α. FLAG (green) and P-STAT1 (red) signals were detected. Nuclei (blue) were stained with Hoechst. Scale bar, 10 μm. (b) Western blot of HEK293T transfected with empty vector (EV) (lane 1–2) or ZIKV NS2A (lane 3–4) stimulated (lanes 2–4) or not (lanes 1–3) with IFN-α. Membranes were stained for P-STAT1, STAT1-tot and GAPDH antibodies. The ratio p-STAT1/STAT1 is showed and represents relative intensity of the p-STAT1/STAT1 detected by Western blot. Quantification of bands was performed to calculate percentage of P-STAT1 and STAT1-tot protein levels relative to control. Significance was calculated using a two-tailed unpaired Student’s t-test, **P< 0.01. Error bars indicate the mean ± SD. Data from at least 3 independent experiments

Previous results showed that NS2A can interfere with the JAK-STAT signaling cascade in response to both types of IFN, causing the suppression of gene expression. As it has been deeply investigated, the induction of type I and type-II IFNs occurs through the activation of a signal cascade which results in the phosphorylation and translocation of transcription factors, including STAT1 [13]. While IFN-α/β stimulation preferentially leads to the formation of a heterodimeric complex with STAT2, IFN-γ stimulation results in the homodimerization of STAT1 [7,13]. STAT1 complexes from cytoplasm are then translocated to the nucleus where they switch on the ISRE and GAS promoter, respectively. Thus, given that the phosphorylation of STAT1 is the mainly step shared in both type-I and type-II IFN response, we firstly examined phosphorylation of STAT1 at Tyr701 and its subcellular localization by immunofluorescence in the presence and absence of NS2A. HEK293T were transfected with empty vector or expression plasmids for NS2A or EBOV VP24, known inhibitor of STAT1 nuclear transport [40]. After transfection, cells were incubated with IFN-α for 30 min and then were double immunostained for FLAG and P-STAT1 (Figure 2(a)). Results showed that in cells transfected with empty vector and stimulated with IFN-α, P-STAT1 was detectable in the nuclei (Figure 2(a), row 2), while as expected in the not stimulated cells no P-STAT1 was observed (Figure 2(a), row 1). Notably, in cells transfected with NS2A plasmid, P-STAT1 was not detectable upon IFN-α stimulation (Figure 2(a), row 3), while in cells transfected with EBOV VP24 plasmid P-STAT1 was observed only in the cytoplasm (Figure 2(a), row 4), consistently with its mode of action. This clearly demonstrated that ZIKV NS2A is acting differently from EBOV VP24, inhibiting STAT1 phosphorylation and not just the P-STAT1 nuclear transport.

To further confirm NS2A-mediated inhibition of STAT1 phosphorylation, NS2A effects were also evaluated by Western blot analysis. HEK293T cells, transfected with empty pcDNA3.1 vector or NS2A expression plasmid, were stimulated with IFN-α for 30 minutes; total proteins were then extracted and levels of P-STAT1 were quantified. As expected, in the whole NS2A transfected cells lysate P-STAT1 levels were significantly reduced as compared to empty vector transfected cells (Figure 2(b)).

Hence, to ascertain whether NS2A might directly inhibit STAT1 phosphorylation, we evaluated the effect of the viral protein on total STAT1 (STAT1-tot) levels. Interestingly, when we stained cells for STAT1-tot, we observed that the STAT1 levels in NS2A cells were certainly lower (~50%) than those in empty vector cells (Figure 2(b)).

**Figure 3.**
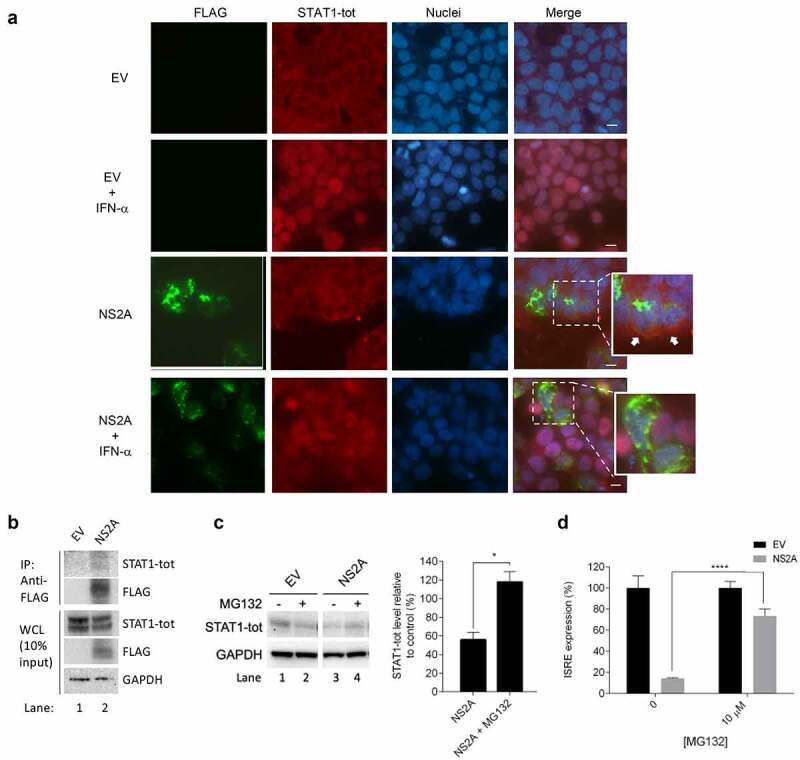
**ZIKV NS2A mediates STAT1 degradation**. (a) Immunofluorescence of HEK293T cells transfected with empty vector (EV) stimulated (row 2) or not (row 1) with IFN-α, or ZIKV NS2A (row 4) stimulated or not (row 3) with IFN-α. FLAG (green) and STAT1-tot (red) signals were detected. Nuclei (blue) were stained with Hoechst. Arrows indicate the colocalization of NS2A with STAT1-tot. Scale bar, 10 μm. (b) Co-Immunoprecipitation of FLAG-tagged NS2A (IP:FLAG) and STAT1-tot. The presence of proteins in whole cell extracts (WCE) was verified by detection with the corresponding antibody. (c) Western blot of HEK293T transfected with empty vector (EV) (lane 1–2) or ZIKV NS2A (lane 3–4). Cells were treated (lanes 2–4) or not (lanes 1–3) with MG132. Membranes were stained for STAT1-tot and GAPDH antibodies. Quantification of bands was performed to calculate percentage of STAT1-tot protein levels relative to control. (d) Percentage of pISRE-luc expression in HEK293T cells transfected with empty vector (EV) or ZIKV NS2A after stimulation with IFN-α and treatment with MG132

Immunofluorescence microscopy was used to assess the possible functional interactions between NS2A and STAT1-tot. HEK293T cells were transfected with empty vector or NS2A expression plasmids. After transfection, confluent monolayers were incubated with IFN-α for 30 min and were then double labeled with FLAG and STAT1-tot antibodies (Figure 3(a)). Non-phosphorylated STAT1 is a constitutive transcription factor expressed in the cytoplasm, even without IFN stimulation. In fact, in cells transfected with empty vector and not stimulated with IFN-α, STAT1-tot was well detectable in the cytoplasm (Figure 3(a), row 1). As expected, after IFN-α stimulation the red signal moved from cytoplasm to the nuclei (Figure 3(a), row 2). As expected, in NS2A transfected and stimulated cells the red signal of STAT1-tot was totally abolished, confirming the strong inhibition of STAT1 phosphorylation (Figure 3(a), row 4). Notably, in NS2A transfected cells not stimulated with IFN-α, cytoplasmic STAT1-tot immunolabeling was found to overlap with NS2A (Figure 3(a), row 3). Secondly, we used coimmunoprecipitation to confirm the NS2A interaction with STAT1. NS2A-FLAG was transfected in HEK293T cells, subjected to anti-FLAG affinity purification, and analyzed by immunoblotting. Of note, the NS2A-FLAG pulldown resulted in endogenous STAT1 recovery, while in the empty vector elute no bands corresponding to STAT1 were observed (Figure 3(b)).

**(C, D)** Significance calculated using atwo-tailed unpaired Student’s t-test, *P < 0.05, ****P< 0.0001. Error bars indicate the mean ± SD. Data from at least 3 independent experiments.

Once ascertained that NS2A could physically interact with STAT1, we asked whether NS2A might induce STAT1 degradation. Hence, we evaluated whether the proteasome inhibitor MG132 could affect the STAT1 levels in NS2A transfected cells (Figure 3(c)). Results showed that, in the presence of NS2A and MG132, STAT1 levels were totally restored, suggesting that NS2A was degrading STAT1 through the requirement of the cellular proteasome system.

We further evaluated the effect of the treatment with MG132 on the NS2A inhibition of the ISRE expression. We cotransfected cells with pISRE-luc and the plasmid encoding for NS2A or the empty vector. We stimulated cells with IFN-α and treated them with MG132 for 4 hours. Then, we harvested the cells and quantified the luminescence (Figure 3(d)). As expected, in empty vector transfected cells, the treatment with MG132 did not affect the ISRE expression, while in NS2A cotransfected cells the ISRE expression was significantly restored after treatment with the protease inhibitor (Figure 3(d)).

### NS2A degrades STAT2

**Figure 4.**
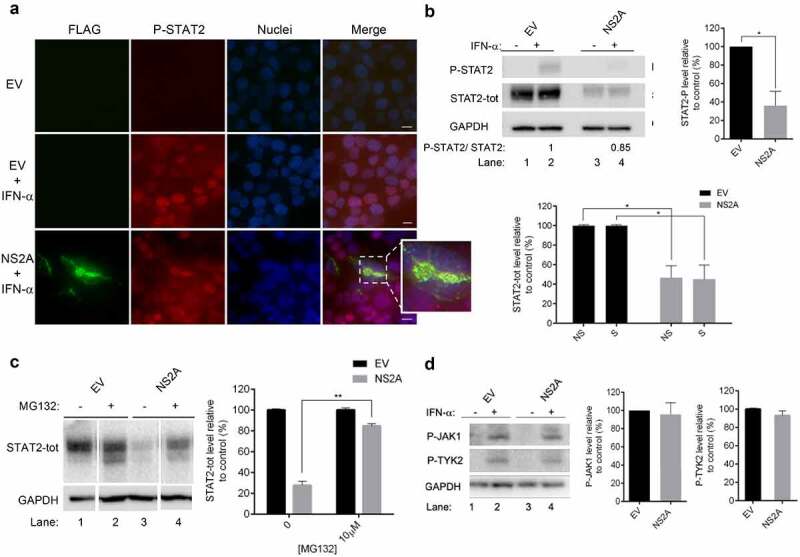
**ZIKV NS2A mediates degradation of STAT2**. (a) Immunofluorescence of HEK293T cells transfected with empty vector (EV) stimulated (row 2) or not (row 1) with IFN-α, or ZIKV NS2A (row 3) stimulated with IFN-α. FLAG (green) and P-STAT2 (red) signals were detected. Nuclei (blue) were stained with Hoechst. Scale bar, 10 μm. (b) Western blot of HEK293T transfected with empty vector (EV) (lane 1–2) or ZIKV NS2A (lane 3–4) stimulated (lanes 2–4) or not (lanes 1–3) with IFN-α. Membranes were stained for P-STAT2, STAT2-tot and GAPDH antibodies. The relative intensity of p-STAT2/STAT2 is showed. Quantification of bands was performed to calculate percentage of P-STAT2 and STAT2-tot protein levels relative to control. (c) Western blot of HEK293T transfected with empty vector (EV) (lane 1–2) or ZIKV NS2A (lane 3–4) treated (lanes 2–4) or not (lanes 1–3) with MG132. Membranes were stained for STAT2-tot and GAPDH antibodies. Quantification of bands was performed to calculate percentage of STAT2-tot protein levels relative to control. (d) Western blot of HEK293T transfected with empty vector (EV) (lane 1–2) or ZIKV NS2A (lane 3–4) stimulated (lanes 2–4) or not (lanes 1–3) with IFN-α. Membranes were stained for P-JAK1, P-TYK2 and GAPDH antibodies. Quantification of bands was performed to calculate percentage of P-JAK1 and P-TYK2 protein levels relative to control

Another important cellular transcription factor involved in the IFN signaling is STAT2, a subunit of the multimeric transcription factor ISGF3, which is recruited in response to type I IFN stimulation. ISGF3 is formed by two STATs, STAT1 and STAT2, in association with a non-STAT protein, IRF9 [42]. To further investigate the inhibition of IFN signaling by NS2A, we firstly evaluated whether NS2A might interfere also with the subcellular localization of STAT2. In fact, NS2A induced STAT1 degradation and the block of its phosphorylation should also prevent STAT2 nuclear translocation, even after stimulation with IFN-α and STAT2 subsequent phosphorylation. To verify such scenario, we probed HEK293T cells with the antibody for P-STAT2 (Figure 4(a)). As expected, no P-STAT2 was present in unstimulated cells (Figure 4(a), row 1), while STAT2 was phosphorylated in IFN-α-stimulated empty vector (Figure 4(a), row 2) as well as in NS2A transfected cells (Figure 4(a), row 3). This result indicates that STAT2 phosphorylation was not suppressed by the NS2A protein. In addition, as expected, in cells transfected with empty vector P-STAT2 had a nuclear localization (Figure 4(a), row 2), while in cells transfected with NS2A, P-STAT2 was localized in the cytoplasm and not in the nucleus (Figure 4(a), row 3). Notably, the immunofluorescence revealed that P-STAT2 colocalized with NS2A (Figure 4(a), row 3). Hence, we asked whether there could be an interaction between NS2A and STAT2. Results showed that no endogenous STAT2 was immunoprecipitated with FLAG-NS2A in a Co-IP experiment (data not shown). To further confirm that NS2A was not affecting the STAT2 phosphorylation, we performed a western blot for P-STAT2 and total STAT2 (STAT2-tot) in cells transfected with empty vector or NS2A. Unexpectedly, we observed a strong decrease in the levels of both P-STAT2 and STAT2-tot, with a comparable percentage, in NS2A transfected cells (Figure 4(b)). We asked whether NS2A could induce the degradation of STAT2. By using compound MG132 (Figure 4(c)), we observed that this compound reduced STAT2 degradation in NS2A transfected cells, as it was observed for STAT1. Finally, we measured the levels of P-JAK1 and P-TYK2, the kinases responsible for STAT1 and STAT2 phosphorylation, respectively, observing that they were not changed in NS2A transfected cells as compared to empty vector transfected cells (Figure 4(d)). This suggested that NS2A is acting only degrading STAT1 and STAT2.

**(B, C)** Significance calculated using atwo-tailed unpaired Student’s t-test, *P < 0.05, **P< 0.01. Error bars indicate the mean ± SD. Data from at least 3 independent experiments.

### Effect of NS2A truncated forms on IFN-signaling

**Figure 5.**
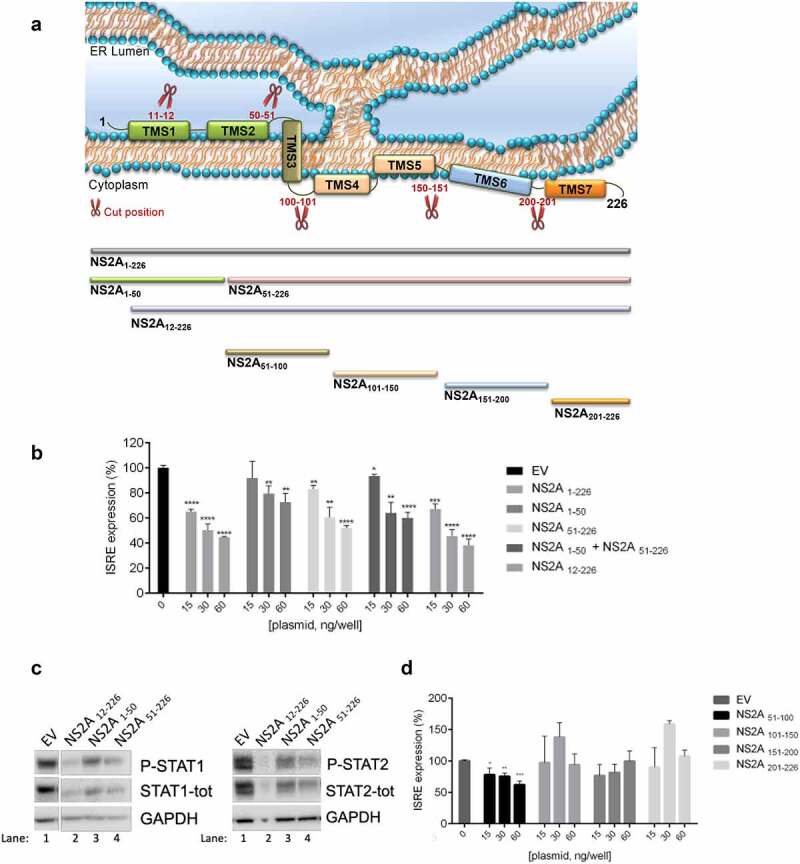
**Effect of NS2A fragments on IFN signaling**. (a) Schematic representation of ZIKV NS2A and fragments with relative cut positions. (b) Percentage of pISRE-luc expression in HEK293T cells transfected with empty vector (EV) or entire ZIKV NS2A_1-226_ and NS2A_1-50_, NS2A_51-100_, alone or together, and NS2A_12-226_ after stimulation with IFN-α. (c) Western blot of HEK293T transfected with empty vector (EV) (lane 1) or ZIKV NS2A_12-226_ (lane 2), NS2A_1-50_ (lane 3), NS2A_51-100_ (lane 4) stimulated with IFNα. Membranes were stained for P-STAT1, STAT1-tot, P-STAT2, STAT2-tot and GAPDH antibodies. (d) Percentage of pISRE-luc expression in HEK293T cells transfected with empty vector (EV) or fragments NS2A_51-100_, NS2A_101-150_, NS2A_151-200_ and NS2A_201-226_ after stimulation with IFN-α

As described in a recent paper by *Zhang et al*. [43], ZIKV NS2A possesses seven transmembrane segments (TMSs): the NS2A 56 amino acid N-terminal residues reside in the ER lumen, the region of residues 74–97 traverses the ER membrane, and the C-terminal region, residues 103–226, is located in the cytosol (Figure 5(a)). Hence, we asked whether the NS2A domain/s required for evasion of IFN-α/β activation was located in the N-terminal or C-terminal portion of the viral protein. We used ProP 1.0 [44] and PROSPER servers to identify possible signal peptide cleavage sites in the NS2A sequence. Both servers predicted a common cleavage site between the position between amino acid residues 50 and 51 (GFS↓MS). Based on this prediction, we hypothesized that probably a NS2A portion could be cleaved to exert its anti-IFN function in the cytoplasm. To investigate this, we subcloned a C-terminal HA-tagged NS2A_1-50_, and a C-terminal FLAG-tagged NS2A_51-226_ into the mammalian expression vector pcDNA3.1 and used the constructs in the previously described cellular system. Interestingly, both truncated NS2A forms were able to inhibit the ISRE expression, but both had lower inhibition efficiency than full-length NS2A (Figure 5(b)). Of note, when both fragments were co-transfected simultaneously, the ISRE expression inhibition was the average of the individual effects (Figure 5(b)) so that, together, they were not able to restore the full-length NS2A inhibition, suggesting that the whole protein was needed to potently act as IFN antagonist. Of note, total inhibition of the ISRE expression was observed when a truncated form of NS2A (NS2A_12-226_), lacking the first 12 N-terminal residues, was used (Figure 5(b)), suggesting that these amino acid residues were not involved in the IFN inhibition. A drastic reduction of P-STAT1, P-STAT2 and total proteins was achieved when cells were transfected with NS2A_12-226_ compared to empty vector transfected cells (Figure 5(c)). Moreover, transfection with the fragment NS2A _51–226_ also results in the strong reduction of STAT1 and STAT2 protein levels, as compared to NS2A _1–50_, in accordance with the percentages of inhibition of the ISRE expression (Figure 5(b)).

**(B-D)** Significance calculated using atwo-tailed unpaired Student’s t-test, *P < 0.05, **P< 0.01, ***P< 0.001, ****P< 0.0001. Error bars indicate the mean ± SD. Data from at least 3 independent experiments.

Next, given that the C-terminal FLAG-tagged NS2A_51-226_ was effective in inhibiting IFN cascade activation, we asked whether single domains of the NS2A_51-226_ protein could be specifically involved in the ISRE inhibition (Figure 5(d)). To this aim, we constructed plasmids encoding fifty amino acids length-NS2A fragments: the construct 51–100 included TMS3, the construct 101–150 included TMS4 and TMS5, fragment 151–200 included TMS6, and fragment 201–226 included TMS7 (Figure 5(a)). To investigate their effect on ISRE expression, we cotransfected the cells with each of these constructs at different concentration and stimulated them with IFN-α. Results showed that fragment NS2A_51-100_ was significantly able to suppress the ISRE expression, while all other fragments did not have any effect on ISRE inhibition (Figure 5(d)). Taken together, these results suggest that the residues localized between position 12 and 100 are necessary for the inhibition of the IFN signaling.

### Comparative analysis of ZIKV NS2A sequence reveal high identity of domain 12-100 among different flaviviruses

**Figure 6.**
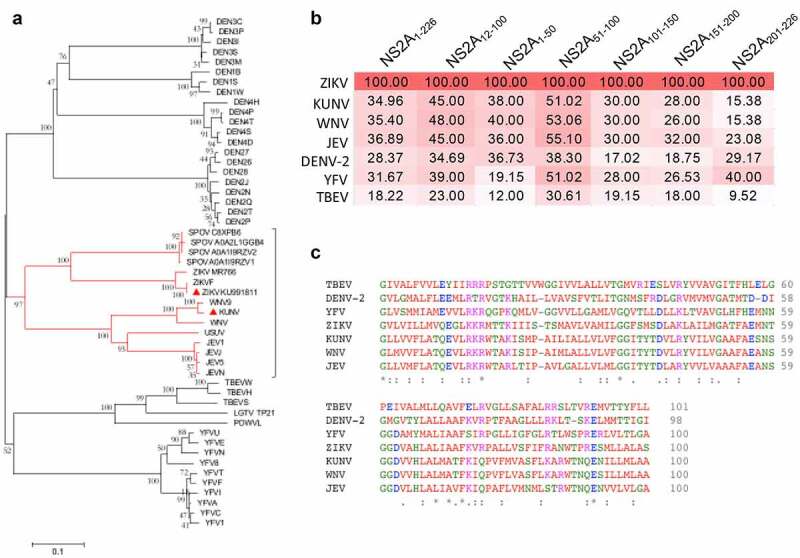
**Phylogenic tree of flaviviral NS2A and identity percentages among NS2A fragments**. (a) Neighbor-joining (NJ) tree based on amino acid sequence of ZIKV NS2A and other *Flavivirus*. In red, it is represented the significant relation (bootstrap value = 97) between ZIKV NS2A with other flaviviral NS2A. Amino acids sequence were obtained from Uniprot. (b) Percentages of sequence identity between NS2A and fragments of ZIKV KU991811 and best representative member of *Flavivirus*. (c) Alignment of ZIKV NS2A_12-100_ and the same NS2A region in other *Flavivirus.*

The phylogenetic analysis of the ZIKV NS2A amino acid sequence and other *Flaviviruses*, supported the strong correlation between ZIKV NS2A and KUNV and WNV showing a 97% bootstrap value (Figure 6(a)). However, the alignment of the NS2A amino acid sequence of ZIKV with the ones of KUNV and WNV showed a low identity (34.96% and 35.40%, respectively) (Figure 6(b)). It was hence possible to hypothesize that this might be explained by the fact that not the entire NS2A protein is involved in the IFN inhibition, as confirmed by single-domain results. To verify this hypothesis, we aligned the NS2A amino acid residues 12–100, the one that we found to be involved with the IFN-evasion, with the same region of the other flavivirus, and observed a high conservation of this domain (Figure 6(c)). In fact, the percentage of identity of this domain was higher than the identity of the entire protein alignment. In particular, KUNV, WNV and JEV NS2A regions possess the highest percentages (45%, 48%, and 45%, respectively). In addition, we aligned the different NS2A fragments used in our study, observing that the percentages of identity increased for the fragment NS2A _51–100_, that shows identity with KUNV, WNV and JEV of 51%, 53% and 55%, respectively (Figure 6(b)). Of note, the fragment NS2A _1–50_ showed an amino acid similarity higher than the one of the entire protein (38% and 40% for KUNV and WNV, respectively), but lower than the one of the NS2A_12-100_ (Figure 6(b)); while NS2A_101-226_ had a very low identity among all *Flaviviruses* (Figure 6(b)). Very low identity was observed also between the NS2A sequences of ZIKV and TBEV and YFV (Figure 6(b)); consistently with the lack of IFN signaling antagonism shown by TBEV and YFV NS2A. Notably, ZIKV NS2A _12–100_, ZIKV NS2A _1–50_ and ZIKV NS2A _51–100_ fragments showed an amino acid identity with the DENV-2 similar fragments (34.69%, 36.73% and 38.30%, respectively) higher than the one observed aligning the entire protein (28%) (Figure 6(b)). Indeed, in spite of the phylogenetical distance between ZIKV and DENV-2, their NS2A are both able to inhibit the ISRE promoter transcription through the reduction in STAT1 phosphorylation [45]. Taken together, these data support the hypothesis that the NS2A amino acid residues 12–100, could play a critical functional role in the efficient evasion of the IFN signaling by ZIKV, KUNV, WNV and DENV-2 NS2A. ZIKV NS2A_12-100_ fragment mostly corresponds to the portion of the protein crossing the ER membrane in a lumen-to-cytosol direction. This result suggests that NS2A remains localized in the ER and therefore its inhibition of the IFN signaling occurs in proximity of the endoplasmic reticulum.

**(B-C)** NS2A sequences used were DENV (sp|P14340|1128-1345), WNV (sp|P06935| 1140–1370), TBEV (sp|P14336|1129-1358), YFV (sp|P03314|1131-1354) and JEV (sp|P32886|1147-1373). NS2A ZIKV and KUNV were from ZIKV KU991811 and KUNV strain MRM61C, relatively.

## Discussion

Dependent on cell type and viral strain, ZIKV infection leads to the induction and/or evasion of the IFN system. ZIKV strains are grouped into two lineages: the Asian and African lineages. Comparison between strains demonstrated that African MR766 strain causes more severe brain damage and newborn fatality than the Asian MEX1–14 strain [46]. Infecting human primary neural cells with African and Asian lineage strains, the African strain induced upregulation of genes including RIG-I, MDA-5, and TLR-3 and induction of type I and II IFN was higher, while the Asian strain did not show any significant upregulation of genes [47]. Infected human blood monocytes are permissive to the Uganda strain MR766 or the French Polynesia strain H/PF/2013 infections. However, the infection with Asian viruses led to the expression of an immunosuppressive phenotype. Different kinetics of replication can be the cause of the difference in IFN induction by diverse lineages [21]. A recent paper described the antiviral response of to MR766 to the strain BeH819015 [48]. They observed that pDCs co-cultured with ZIKV-infected cells triggered a barely detectable IFN-α response and no production of inflammatory mediators, in contrast with previous studies conducted with related flaviviruses [48–51]. Also the Asian INMI1 ZIKV strain was reported to not induce both type I and type III IFN while poorly activates type II IFN in PBMC [52]. Such slight modulation of type II IFN and the complete lack of type I/III IFN induction suggests the ability of ZIKV to evade the IFN system [52].

The non-structural proteins of ZIKV are known to be responsible for suppression of IFN production and its downstream signaling. Among them, NS2B3 of MR766 strain has been demonstrated to block the IFN pathway by degrading human STING, while the NS2B3 of the Asian Z1106033 strain has been shown to suppress the IFN-activated signaling by cleaving JAK1 [24,53]. Furthermore, a single-point mutation (A188V) in NS1 protein of FS13025 strain enables the virus to inhibit the IFN production [23] and NS4A of the FS13025 strain impairs the interaction between MAVS and RIG-I [54]. NS5 of MR766 strain is known to bind STAT2 for degradation [26], the NS5 of KU527068 strain can block STAT1 phosphorylation [55] and the NS5 protein of an Asian strain (H/PF/2013) inhibits IFN production [22,23]. Finally, NS2A, NS2B, and NS4B of the Asian FSS13025 strain inhibit the IFN production via inhibition of TBK1 activity [23].

Here, we revealed a mechanism adopted by the Asian INMI1 ZIKV strain to hinder the IFN signaling cascade. Our results demonstrated that the expression of ZIKV NS2A resulted in the dose-dependent inhibition of the IFN-α-induced ISRE transcription in the dual-luciferase assay as well as of the transcript levels of the ISGs mRNA ISG15 and OAS1. Mode of action studies showed that ZIKV NS2A antagonized type I IFN-mediated phosphorylation of STAT1 and STAT2 promoting the STAT1 and (iii) STAT2 degradation.

The inhibition of the IFN signaling has been described for other *Flaviviruses*. However, the mechanisms by which most of these viruses block the IFN signaling are not completely understood. Infection with both KUNV (a WNV variant) and NY99 strain of WNV prevents the translocation of STAT1 and STAT2 from the cytoplasm to the nucleus by inhibiting the STAT1 and STAT2 phosphorylation in response to IFN treatment, and it is also able to downregulate STAT1 expression [56]. Indeed, KUNV NS2A was observed to block P-STAT2 nuclear translocation without inhibiting its phosphorylation [56], showing a mode of action partially similar to what we observed for ZIKV NS2A.

**Figure 7.**
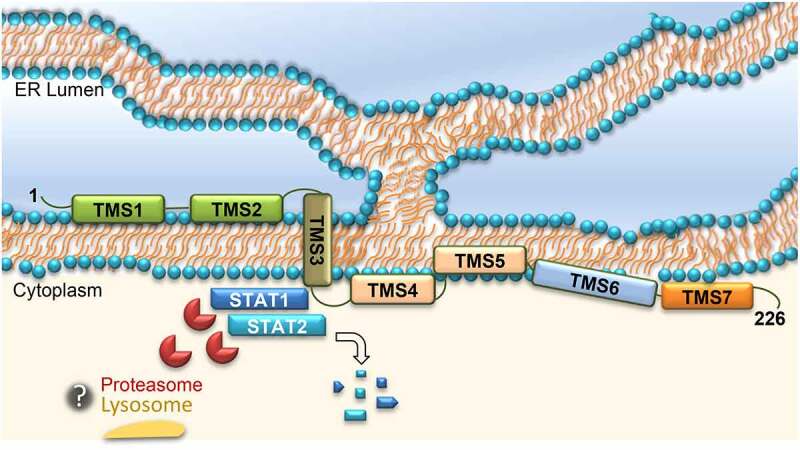
Schematic representation of STAT1 and STAT2 degradation mediated by ZIKV NS2A

Taking into consideration the NS2A ability to degrade both STAT1 and STAT2, it is not surprising that a single protein acts by degrading both transcription factors. Indeed, the degradation of STAT1 and STAT2 by a unique protein has been observed for different viruses, such as various members of the family *Paramyxoviridae*. Studies on Mumps virus provide genetic evidence of a degradation factor that is composed minimally of STAT1, STAT2, and V protein. As we observed for ZIKV NS2A, the degradation of STAT proteins by Mumps virus has been proposed to be the result of the ubiquitin-proteasome complex, as it could be also inhibited by MG132. Hence, it is tempting to speculate that NS2A, as protein V, could form a functional NS2A-STAT1-STAT2 protein complex, which recruits the cellular proteasome to promote degradation of critical transcription factors, responsible for the ISGs expression, thus suppressing the IFN signaling (Figure 7).

Furthermore, it was recently reported that ZIKV-induced KPNA2 reduction is due to the lysosomal proteolysis as treatment with both MG132 and NH4Cl restores the KPNA2 level in ZIKV-infected cells and that such effect it is due to NS2A [57]. In our study, we demonstrated the effect of MG132 in restoring the levels of STAT1 and STAT2. MG132 is often used to assess the involvement of the proteasome system in the virus induced-protein degradation [58]. Given that it also inhibits some lysosomal proteases [57], our results do not completely exclude the possibility of the involvement of the lysosomal proteolytic pathway in the degradation of both transcription factors. However, while the ubiquitination of STAT1 and STAT2 signaling proteins and their subsequent degradation in the proteasome system is the common and more frequent strategy adopted by several viruses, including Flavivirus, to shut down the innate immune response [26,58–62], the virus‐induced autophagic degradation of STAT1 and STAT2 as a mechanism for interferon signaling inhibition has been reported only by few papers [63,64]. For instance, *Rojas et al*. reported that STAT2 was degraded through autophagy/lysosome pathway by NS3 of Bluetongue virus. However, the treatment with MG132 did not restore the expression of STAT2 [63] (Rojas et al., 2019). Another recent study suggests that SARS-CoV-2 M^pro^ might prompt the autophagic degradation of STAT1. Also, in this case, MG132 was not able to rescue the level of STAT1 [64]. Hence, we think that the pathway of the proteasome degradation might be the preferential one adopted by ZIKV NS2A, even if – again – we cannot exclude neither the involvement of the lysosomal pathway, nor a cooperation between the two systems.

Our results suggest that the domain mainly involved in the NS2A function of IFN signaling inhibition is included between amino acids 12 and 100. Functional analysis has defined distinct NS2A residues essential for viral RNA synthesis and replication [43]. It is important to underlie that six out of nine NS2A mutants (K56A, F83A, K84A, R86A, P87A, R96A) that were found to dramatically reduce ZIKV replication, are within the region we identified to be involved in the IFN antagonism. Moreover, ZIKV virus with NS2A G12A substitution was completely defective in viral RNA synthesis. These data suggest that mutations in residues involved in the IFN signaling might be responsible for the attenuation of viral replication. Further studies would be required to investigate how these mutations affect NS2A anti-IFN efficacy.

For viruses such as ZIKV that adopt evasion mechanisms to overcome the cellular immunity, the restoration of the IFN response is an attractive therapeutic approach to control the infection. Several recent studies have proved valuable the strategy of recovering the IFN system by targeting viral anti-IFN proteins [32,34,65–69] and/or upregulating the host’s own natural immune response [70–72]. For example, DENV NS4B, in virtue of its efficient suppression of the host IFN defense, has been deeply investigated as a potential antiviral target [69]. Moreover, recent studies support the use of antiviral development strategies based on the upregulation of the IFN response against ZIKV infection [71–73]. An example it is the treatment with azithromycin that blocks ZIKV replication by enhancing the expression of several ISGs [74]. While ZIKV NS2A has not been used as an antiviral target in any drug-development study to date, our evolving knowledge of its multiple roles in host immune-response evasion makes it a viable possibility. Given the critical role of IFN system in prevention of ZIKV infection, these studies will be of great value for future identification of compounds directed against a novel therapeutic target like NS2A.

## Data Availability

The authors confirm that the data supporting the findings of this study are available from the corresponding author [E.T.].
